# Manual of International Classification of Causes of Death

**Published:** 1903-07

**Authors:** 


### Manual of International Classification of Causes of Death.

THE JOURNAL has received from the chief statistician of the American census office a document with the foregoing title. It is accompanied by a number of circulars in pamphlet form, the purpose of these several brochures being to promote a uniformity of registration of vital statistics, "in order to make the census mortality statistics more valuable."

This incident gives us the opportunity to remind oui readers that vital statistics are the gold and silver, the coin of the realm of hygiene; that in coins accuracy and purity are of supreme importance, and in vital statistics causes of death must be in terms of exactness; that facts must be stated not only with preciseness, but in terms having exact value. All this is necessary that the mind of the observer who writes the certificate may be plainly understood by the local health officer, by the health officer at the capital of his state, by the government at

Washington, and everywhere over the entire world where use is made of vital statistics.

We wish this manual might find its way into the hands of every man authorised by law to make a death certificate, and we advise that it be studied and consulted by all such to make sure that causes of death shall only be stated in authorised official terms. Our local health officer will find this manual authority for returning many certificates to the writers with the suggestion that the cause of death is too indefinite for the purposes of his office. Town local health officers will find,—and would do well to take advantage of such finding,—in this manual a nomenclature from which they can improve their own publications.

This manual has been adopted by the census office at Washington for the compilation of mortality statistics, beginning with the year 1900. It contains by far the most comprehensive, most complete, and most scientific nosological tables that have yet been published in America. Moreover, it is replete with instructive suggestions, and merits the careful examination, if not study, of every person interested in the preparation of vital statistics.

America is to be congratulated upon this important step for the advancement of state medicine, and we ask in its support the united efforts of the medical profession.

---

ALL physicians will rejoice over the success of the Child Labor Committee of New York, in securing the passage of bills by the last legislature regulating the employment of children. The *Buffalo Express* of June 18, gives a summary of the law as follows:

The perjury of parents regarding the age of children under fourteen years old in order to secure employment for them in factories and stores has been made impossible by requiring as evidence of age either a transcript of the child's birth or baptismal certificate, or some other religious record, or its passport; the clause of the old law allowing children twelve to fourteen years old to work in stores during vacation has been repealed for all cities of the first and second class; a nine-hour limit has been placed upon the work of children fourteen to sixteen years old in factories and stores, in place of a ten-hour limit; the employment of messenger, telegraph, delivery and office boys less than fourteen years old has been forbidden, while between the ages of fourteen and sixteen years they may be employed only nine hours a day and not later than 10 o'clock at night; no boy less than ten years old is allowed to sell newspapers in New York and Buffalo and all boys between the ages of ten and fourteen years must receive licenses or badges and may not work later than 10 o'clock at night; the compulsory school-attendance age has been raised from twelve to fourteen years:

There is no doubt that the interests of health, intelligence and morality among the young are served by these measures, provided the enforcement of the laws can be secured; and to this end funds are solicited which may be sent to the Child Labor Committee, V. Everit Macy, No. 68 Broad Street, New York.

---

THE New York State Nurses's Association has been instrumental in securing the passage of a bill which provides a system of registry for regularly trained nurses who shall have passed a satisfactory examination before a board of examiners appointed by the regents of the University. The association, under the provisions of the bill, is to nominate a list of ten members, from which the examiners are to be selected. Nurses passed by the board each will receive a license as registered nurse, and may attach R. N. to their names, as may also nurses already qualified by long service. The bill became a law April 27, 1903, the day it was signed by Governor Odell.

---

DR. JOHN P. BRYSON, one of the foremost genitourinary surgeons in this country, died at his home in Saint Louis recently, after a brief illness. Dr. Bryson was graduated from the Humboldt Medical College in 1868, and at the time of his death was genitourinary surgeon in the medical department of Washington University and surgeon to the Saint Louis Mullanphy Hospital. He was a member of the American Association of Genitourinary Surgeons and the Saint Louis Medical Society. He was one of the founders of *American Medicine*.

Dr. Bryson's death will be keenly felt in Saint Louis, where he took an active interest in civic affairs. His chief work as a professional man was in genitourinary surgery, and he ranked high as an operator on the prostate. His writings on this subject and his description of the prostate and its diseases and abnormalities are classics and will long remain unequalled for lucidity and scientific accuracy. Especially is this so of one of the last papers Dr. Bryson prepared, that which he read before the meeting of the American Association of Genitourinary Surgeons at Atlantic City, in May of last year, on The technic of prostatectomy. In this paper he compared the suprapubic and perineal routes and exhibited specimens, some taken as far back as 1895, to prove his contention that the suprapubic route was the proper method. Another paper, entitled, A possible aid to the discovery of the tubercle bacilli in urine, read in Washington in 1897, remains to this day one of the most important contributions to the subject which has been issued.

